# Healthcare professionals’ perceptions of interprofessional teamwork in the emergency department: a critical incident study

**DOI:** 10.1186/s13049-022-01034-0

**Published:** 2022-07-15

**Authors:** Jenny Milton, Annette Erichsen Andersson, N. David Åberg, Brigid M. Gillespie, Lena Oxelmark

**Affiliations:** 1 Institute of Health and Care Sciences, The Sahlgrenska Academy, University of Gothenburg, Arvid Wallgrens backe, hus 1, 41346 Gothenburg, Sweden; 2 Gothenburg Emergency Medicine Research Group (GEMREG), Sahlgrenska University Hospital, Gothenburg, Sweden; 3 Department of Orthopedic Surgery, Region Västra Götaland, Sahlgrenska University Hospital/Mölndal, Gothenburg, Sweden; 4 Department of Internal Medicine and Clinical Nutrition, Institute of Medicine, The Sahlgrenska Academy, University of Gothenburg, Vita stråket SU, 41345 Gothenburg, Sweden; 5 Department of Acute Medicine and Geriatrics, Region Västra Götaland, Sahlgrenska University Hospital, Gothenburg, Sweden; 6 Gold Coast University Hospital, Gold Coast Health, Southport, Gold Coast, Australia; 7 School of Nursing and Midwifery & NHMRC Centre of Research Excellence in Wiser Wound Care, Griffith University, 170 Kessels Road, Brisbane, QLD 4111 Australia

**Keywords:** Interprofessional teamwork, Communication, Patient safety, Emergency department, Critical incidents

## Abstract

**Background:**

Interprofessional teams contribute to patient safety during clinical care. However, little is known about how interprofessional teams manage and cope with critical incidents in the emergency department (ED). Therefore, the study aimed to describe healthcare professionals (HCPs) perceptions of critical incidents linked to the enablers of and barriers to interprofessional teamwork in a high-risk setting, the ED.

**Methods:**

Individual interviews with HCPs regarding events at the ED were held during the period of May 2019–January 2020. The Critical Incident Technique approach was used to guide the interviews and the qualitative analysis. Data were analyzed inductively using qualitative content analysis.

**Results:**

Interview participants (n = 28) included 7 physicians (25%), 12 registered nurses (43%), 7 nurse assistants (25%) and 2 administrators (7%). Overall, 108 critical incidents were described. Eight categories that described functional and dysfunctional experiences within interprofessional teamwork were identified: salience of reflection; professional experience makes a difference; demanding physical and psychosocial work environment; balancing communication demands; lacking management support, structure, and planning; tensions between professional role and responsibility; different views on interprofessional teamwork; and confidence in interprofessional team members.

**Conclusion:**

Findings of this study indicate that poor ED-specific communication and limited professional experience are essential factors in handling critical incidents related to interprofessional teamwork. An important aspect of critical incident management is the ergonomics of the physical work environment and how it enables interprofessional teamwork. This study emphasizes the factors enabling interprofessional teamwork to manage critical incidents in the complex working environment of the ED.

**Supplementary Information:**

The online version contains supplementary material available at 10.1186/s13049-022-01034-0.

## Background

Interprofessional teamwork has been identified as a crucial component in the process of ensuring patient safety [1]. However, the concept of teamwork differs between professional disciplines, potentially entailing risks to patient safety, ineffective care, and a dysfunctional work environment [2–4]. Recent research claims that healthcare professionals (HCPs) can benefit from information about the team's own conceptualization of teamwork and its consequences for everyday practice [2]. Disparate perceptions of teamwork among HCPs and management can result in inappropriate teamwork, leading to a dysfunctional work environment. Lyubovnikova et al. [5] have shown a relationship between real team membership (i.e., the team has shared objectives, engage in team reflexivity, and show structural interdependence) and lower patient mortality. Healthcare delivery by dysfunctional teams may undermine interprofessional communication and increase the frequency of errors, both of which have been shown to be associated with adverse events and patient mortality [5].

The emergency department (ED) is considered to be a high-risk environment in which interprofessional healthcare teams need to work together. In this sense, ED care has been compared with other high-risk operations, such as those in the aviation and nuclear industry sectors, where minor errors can have devastating consequences for humans, equipment, and the environment [6, 7]. Indeed, dysfunctional teamwork and communication are considered to contribute to adverse events [6, 8] in both the ED [9] and general healthcare system [10]. Human errors, which are the most frequent contributing factors, comprise interactions between humans and ergonomics, processes, technical design, communication and leadership [11]. In fact, the US Joint Commission for the Accreditation of Healthcare Organizations has reported that human factors (i.e., the relationship between human performance and the environment safety) contribute to > 70% of all adverse events in healthcare [12]. Similarly, a national report on preventable adverse events in Swedish somatic healthcare [13] has highlighted patient safety in relation to human communication*.*

It has been suggested that the organizational structure contributes to adverse events through deficits in leadership, staff planning, workspaces/layouts, environmental factors, and other aspects [6]. Other factors, often intangible, include the importance of informal communication, as shown for situations involving bedside assessments and opportunistic interactions [14]. HCPs’ experiences of interprofessional teamwork in the ED can guide a better understanding and improvement of interprofessional teamwork.

To our knowledge, little research has been performed on interprofessional teamwork and communication skills in the context of critical incidents in the ED. Thus, it is essential to identify the complexity of the functions and roles of the different professions [15] needed to achieve safe care [2]. Therefore, we aimed to describe HCPs’ perceptions of critical incidents linked to enablers of and barriers to interprofessional teamwork in the ED setting. The following research questions were addressed: (1) How is clinical information described as communicated within the team?; (2) What are the perceived enablers of interprofessional teamwork?; and (3) What are the perceived barriers to interprofessional teamwork?

## Methods

### Design

This study was designed to describe enablers of and barriers to interprofessional teamwork using interviews with HCPs according to the critical incident technique (CIT) [16]. An incident is described as any human activity that is significant for gaining an understanding of the persons involved in the act. An incident is defined as critical when the situation is crucial in terms of its consequences and effects. Moreover, the critical incident must entail real life experiences described by the participant [16]. The CIT is appropriate as a qualitative method to describe behaviors based on individual perspectives and known to be an effective clinical tool within healthcare and treatment [17]. The study is reported according to the consolidated criteria for reporting qualitative research (COREQ) guidelines [18] (Additional file 1).

### Research setting

The interviews were conducted in the ED of a university hospital with a catchment area of 1.7 million residents for specialist referrals and approximately 200,000 for primary referrals. The study ED admits patients over the age of 16 years with various medical and surgical conditions and is the regional center for treating trauma and casualties. In 2019, when the interview data were collected, the ED recorded approximately 58,000 patient presentations per year. All visiting patients either entered the ED via pre-hospital transport care or requested care at their own discretion. In both cases, an interprofessional team assessed the patients through: 1) a designated triage space; or 2) transition to one of the assessment team pods (i.e., medical, surgical, and trauma) within the ED, before possible admission to the hospital. Based on pre-arrival contacts between the ambulance and specialized senior doctors, some patients bypassed the ED and were admitted directly to a hospital ward, i.e., patients with certain acute myocardial infarctions and strokes, and some pre-assessed geriatric patients. At the ED, interprofessional teams were present in all the team pods and included a physician, a registered nurse and a nurse assistant, although not all members of the team were present throughout the patient examination. One physician was designated as medically responsible for the entire ED (*ED lead physician*), and another physician was designated as the ‘trauma leader’. Alongside the ED lead physician, there was a registered nurse, who was designated as being responsible for nursing care (*ED lead nurse*) and coordination of the nursing staff. Few of the registered nurses and nurse assistants were specialists in emergency care. However, all the nursing staff was given in-service specialized training in acute and emergency care.

### Participants

The participants were HCPs from the ED, including registered nurses, physicians, administrative staff, and nurse assistants. The nursing staff participants had levels of formal emergency specialist care and in-service emergency care. The physician specialties were emergency medicine and internal medicine, and the level of experience ranged from interns to resident physicians and consultants. The administrative staff were in-service trained to carry out administrative tasks and part of the interprofessional teamwork. In total, 34 eligible participants were identified from the larger group to participate in the study and were contacted via e-mail to schedule an oral interview. Six of these declined due to time constraints, resulting in a final total of 28 participants. The study information and the interview questions were sent via e-mail, two days prior to the interview.

### Data collection

Data collection was performed during autumn 2019, according to the CIT strategies [16]. The goal was to describe HCPs’ perceptions of critical incidents associated with interprofessional teamwork in the ED. A ‘critical incident’ in this study was defined as a situation that made a significant contribution to the general aim of the study, i.e., to describe HCPs’ perceptions of critical incidents linked to enablers of and barriers to interprofessional teamwork, in line with the originally described negative or positive aspects of a scenario [16]. Purposive data sampling was performed to target maximum variation with respect to professional role, gender, age, and years of experience [19]. The only exclusion criterion was if a HCP was recently (< 1 month) employed in the ED or was undergoing an introduction program with supervision.

Interviews were performed face-to-face (*n* = 26) or via telephone (*n* = 2), whichever was more convenient for the participant [20]. The clarity and understanding of the questions were tested through five pilot interviews, one of which was included in the study. Minor changes were made to the interviewing guide and developed according to the lead words “hinder and possibilities” supported by Flanagan’s interview guide and critical questions about finding a strong negative or strong positive reaction to the studied phenomenon [16]. As the interviews progressed, modifications of the CIT guidelines according to Fridlund et al. [17] were used. Consequently, the main question as to ‘critical incident’ was synonymously replaced with ‘a specific event’, in the Swedish translation, as this would better explain and identify the information sought [15] (English translation in the Additional file 1). The first author (JM) conducted and recorded all 28 interviews, each of which lasted 30–45 min. The participants knew the first author as a post-graduate nurse who was undertaking doctoral studies and conducting research in the studied ED. In addition, the author had no other connection to the work environment or the participants that might have influenced the participants’ responses [19]. Data saturation was considered to be reached when no new information emerged [21]. All the interviews were digitally recorded and verbatim transcribed. The transcription was carried out by the first author (JM) (*n* = 8) and a professional transcription agency (*n* = 20).

### Ethical considerations

The participants were informed about the research study through staff meetings, e-mail newsletters, and letters placed in individual mailboxes at the workplace. Participants were informed about the voluntary nature of their participation, measures to ensure confidentiality, and that their consent to participate could be withdrawn at any time. Informed consent was obtained from all participants in connection with the interviews. Data was stored in a password-protected computer (for electronic data) and in a locked filing cabinet located in the researcher’s office (for paper and interview data). The material was coded and presented in an anonymized way to ensure that individuals could not be identified. This study was approved by the Swedish Ethical Review Authority (D.nr. 363-15).

### Data analysis

An inductive content analysis was used to analyze the textual interview data [22]. The material was read and reread by two authors (JM, LO), to identify and extract critical incidents [17] that would either hinder or enable interprofessional teamwork.

The analysis was conducted in three phases: (1) reading through the material (JM and LO) to identify critical incidents, while AEA, NDÅ, and BG verified the analysis. The incidents were grouped according to their similarities concerning experiences of interprofessional teamwork; (2) the material was organized through an inductive approach, and an open coding was used [22]; and, (3) abstraction of the coding was reported through subcategories and categories. The extracted categories and subcategories were discussed intensively until a final consensus was reached. Management of the data was carried out using the NVivo Pro ver. 12 software (QSR International Pty Ltd., Melbourne, Australia).

### Rigor

The authors of this study contributed with different expertise, supporting the credibility of the findings [19]. Several of the authors are experienced researchers (AEA, NDÅ, BG and LO) in the field of qualitative methods (AEA, BG and LO), while only one practices clinically in the ED (NDÅ).

The research group adopted a reflexive approach during the analytic process, to ensure the trustworthiness of the data. Four of the authors (JM, AE-A, NDÅ and LO) met regularly to discuss the patterns and to test the conclusions for the labeled categories [23]. Consensus was sought through discussion and questioning the patterns of the data [24], and going back and forth between the categories and coding to ensure that the data maintained a close connection to the abstraction [23]. To ensure transferability, the selection of participants was based on heterogeneity of age, gender, role and experience. To strengthen the arguments further, an explanation of the context was sought to gain a better understanding of the studied phenomena [25].

## Results

Four different professions were interviewed, represented by 28 individuals (Table 1). From these shared experiences, a total of 108 critical incidents was identified. The first analysis phase resulted in a median of four (range 0–6) identified critical incidents per participant.. These were further grouped into experiences concerning the critical incident.

**Table 1:** Characteristics of the participants (*n* = 28)

Profession	*n* (%)
Physician	7 (25)
Resident^a^/Intern	4/1
Consultant^a^	2
Registered Nurse	12 (43)
Specialist^a^	2
Nurse assistant	7 (25)
Specialist^a^	3
Administrator	2 (7)
Sex, female	19 (68)

	Median (range)
Age, in years	31.0 (21.0–57.0)
Experience in profession, in years	5.0 (0.92–32.0)

^a^Specialist in emergency medicine and internal medicine for physicians/specialist in emergency care for nursing staff

The most frequently described experience of a critical incident was related to the “ways of communication that enabled interprofessional teamwork” (32%). Experiences related to “teamwork organization-related functions and routines” and “the importance of support from colleagues” each accounted for one-fifth of the critical incidents (20% and 19%, respectively) (Table 2).

**Table 2:** Types of critical incidents associated with interprofessional teamwork in the ED

Type of critical incident	Frequency	
	Critical incidents *n* (%)	Interviews^a^ *n* (%)
Ways of communication that enabled interprofessional teamwork	34 (32)	18 (64)
Teamwork organization-related functions and routines	22 (20)	15 (54)
The importance of support from colleagues	21 (19)	16 (57)
Lack of interprofessional communication	10 (9)	8 (29)
Theoretical and clinical professional practice crucial for interprofessional teamwork	10 (9)	8 (29)
Simulation practice and reflective learning	6 (6)	5 (18)
Resignation after teamwork failure	5 (5)	5 (18)
	108 (100)	28 (100)

^a^Number of interviews that included the type of critical incident. The percentages refer to the total number of critical incidents (*n* = 108, left column), and to the total number of interviews conducted (*n* = 28, right column)

The second and third phases of the analysis generated eight main categories and associated subcategories (Table 3). The described experiences within these categories and subcategories reflected both enablers of and barriers to interprofessional teamwork. From the summary of the categories and subcategories in Table 3, supporting quotes are presented in Table 4.

**Table 3:** Categories and subcategories derived from the qualitative content analysis

Categories	Enablers of interprofessional teamwork in the ED	Barriers to interprofessional teamwork in the ED
	Subcategories	
Salience of reflection	**Self-awareness to scrutinize oneself** Awareness of individual limits was used as a motivation to improve and maintain competency **Team reflection as a way of interprofessional learning** Sharing perspectives of the same experienced situation provided awareness of the team capacity and motivation	**Self-awareness to scrutinize oneself** Lack of ability and opportunity to engage in self-reflection caused HCPs to exceed their limits or use to much self-critique **Team reflection as a way of interprofessional learning** Deprioritizing reflection within the team resulted in a failure of interprofessional teamwork
Professional experience makes a difference	**Experience is a crucial component of professional practice** HCPs who had adequate academic and clinical experience to manage teamwork and patient assessment **Expectation of professional experience** Explicit expectations allowed interprofessional teams to adapt to a situation **Need for continuous development and training** Clinical education and simulation training led to stringency with respect to professional roles and the concept of teamwork	**Experience is a crucial component of professional practice** Lack of experience (academic and clinical) hindered the teamwork and delayed the patient assessment **Expectation of professional experience** A preconception of professional experience had a negative impact on interprofessional teamwork **Need for continuous development and training** Lack of professional and clinical training led to misunderstandings and failure to carry out the work
Demanding physical and psychosocial work environment	**The physical work environment** Positive aspects of being physically close	**The physical work environment** Consequences of alarm fatigue, small assessment space and chaotic environment **Dealing with emotions related to stress** Stress-induced unpleasant behaviors during interprofessional teamwork and concealed emotions
Balancing communication demands	**Applying communication (tools, climate, and attitudes)** The correct use of communication tools and the opportunity to work in an enabling team climate encompassing positive attitudes **Art of concise and clear information** The ability to address the intended information to the appropriate colleague, at the right time **Silent communication** Non-verbal behavior complemented or sometimes replaced verbal communication	**Applying communication (tools, climate, and attitudes)** Inadequate use of communication tools and unpleasant attitudes led to teamwork failures
Lacking management support, structure, and planning		**ED considered an unsuitable place of care** Patients received medically advanced treatments for which the HCPs lacked familiarity or had insufficient space, time or equipment in the ED **Mismatch of available resources and excessive workload** Shortage of resources **Discordant views on strategies of care** Differences of opinions across wards between management and staff with respect to the use of strategies and the focus of care as part of the interprofessional teamwork
Tensions between professional role and responsibility		**Gender roles and hierarchies of expertise** Positioning and disagreements (gender norms and other hierarchies) **Violation of personal and professional integrity** Disrespect for professional expertise and overstepping boundaries
Different views on interprofessional teamwork	**Personal relations and favoritism** Strong personal relationships with team members contributed to trust and psychological safety **Perspective on teamwork attributes** Clear interprofessional team goals and routines (i.e., leadership) guided the interprofessional team forward	**Inadequate involvement/intrusion by the patient** Patients either interrupted the team or overheard inappropriate information **Personal relations and favoritism** Favoring colleagues based on personal relations and appreciation led to disrespect for others in the interprofessional team **Perspective on teamwork attributes** Lack of defined routines, purposes, and descriptions led to discrepant perceptions on how to carry out the interprofessional teamwork
Confidence in interprofessional team members	**Joint team assessments** Additive value of the different areas of expertise generated a comprehensive assessment of the patient **Mutual need for interprofessional support** Sharing vulnerability and emotional dedication in the interprofessional team led to an increased feeling of being supported	**Joint team assessments** Disregarding professional boundaries led to failed interprofessional teamwork, duplication of work, and work overload **Mutual need for interprofessional support** Frustration with unsupportive colleagues

ED, emergency department; HCP, healthcare professional

**Table 4:** Categories, subcategories and quotes from the qualitative content analysis

Categories	Subcategories	Quotes
Salience of reflection	Self-awareness to scrutinize oneself	IP 19: “The demands increase. You have higher expectations of yourself. Okay, I must know this, I must check this. So, for something that might happen in the ED – I feel I should know how to deal with it, especially if I am working alone”. (registered nurse)
		IP 18: “Yes, I was very numb, as I thought it was very difficult situation. I disliked attending emergency calls for a while afterwards. It was probably because we didn’t get to process it properly with debriefing afterwards because that never happened… It was more that one witnessed this person die. Observing that person going from being talkative and alive one minute to being dead the next”. (nurse assistant)
	Team reflection as a way of interprofessional learning	IP 7: “So, she stands there and massages his heart [cut open thorax] and you see how the blood is just spurting out, and then the thoracic surgeon comes over and starts to stitch. And there I am standing with blood all over the place, and everyone wades in the patient’s blood. His blood, you know. […] Yes, I never forget that boy. We can’t even go and sit down somewhere as we are so few. If we who attended the trauma alarm leave, then the staff would disappear out onto the floor. So we have to [deep sigh] you know, change our scrubs and clean our shoes and blow our noses [emotional snorting].[…] We try [to talk] whilst cleaning but we know that we must soon go out again. In the worst case, there will be a new trauma alarm. So you have to act quickly. And when we got out [from the trauma room] it felt crazy”. (nurse assistant)
Professional experience makes a difference	Experience is a crucial component of professional practice	IP 6: “The team did signal but I didn’t really listen and as a result it became really stressful. A super-stressful situation […] But they had actually already informed me that I should go see the patient, since it is so much easier for me than for the intern to look at the patient and assess their need for further care. It is crucial to act sooner rather than later […] In any case, I think that the others in the team acted properly […]. I consider that I was relatively inexperienced as the medically responsible physician and that I was stressed because of that”. (physician)
	Expectation of professional experience	IP 19: “‘Is there any physician here’?! And a resident comes running and says ‘I have not handled any acute situations, so this will be my first.’ And I look at her and say ‘Oh my God, I haven’t responded to a call either, so this will also be my first acute situation.’ So both of us were slightly panicked but we thought ‘Okay, we will simply do the best we can.’ […] Both of us were new and didn’t want to miss anything. So, we double-checked with each other all the time: ‘Okay, you want me to give this now, I will give this now’. And you don’t want to make any mistakes. So it wasn’t that she [the physician] had any expectations that I would do things according to a certain routine, in the way it should be, but rather we talked to each other: ‘Take this, do that’. I think if you are a trauma-trained registered nurse, then you already know what to do without the physician really needing to say much […] It was very calm and nice [giggle], which was good. So it felt good in any case to have experienced an acute situation that was handled successfully”. (registered nurse)
	Need for continuous development and training	IP 18: “I am convinced that training is the ‘A to Z’ actually. You talk a lot about what you do, and there is a lot of simulation training. In addition, you already have a structure, so that with these cases or whatever the situation you should know what to do”. (nurse assistant)
		IP 21: “I did not have any introduction. I only had 1 h, then I was directed to the changing room [ready to start work]”. (physician)
Demanding physical and psychosocial work environment	The physical work environment	IP 6: “We must behave in such a way that we have space to move and think. So, what I have done several times after them [the patients] becoming ill in small rooms, is to move to an acute emergency room. Because it is also that this has a signal value for the staff”. (physician)
		IP 9: “There is too much squawking and fuss and unnecessary twaddle about things that concern the clinical work […]. Because a quiet work environment is much better than a loud screeching one. It would make a difference. It should be organized so that the environment becomes more discrete and calmer. I don’t think that it is possible do so, therefore the focus of the work should be in getting patients out of the ED or not even transferring them into this small ED”. (registered nurse)
	Dealing with emotions related to stress	IP 2: “[…] I know that bad things happen. Partly [because of] the choice of profession, I would never have chosen to be in the ED if I could not accept that it was possible to do something wrong. So it is probably a personality trait. […] I think it’s very sad [describing patient hazard] but I know that it happens and that one must learn from it and move on, because it is part of the job”. (physician)
Balancing communication demands	Applying communication (tools, climate, and attitudes)	IP 11: “For my part, I am active in communicating, in that I am listening to what they are saying and keeping up with what is going on. Therefore, there doesn’t need to be chat all the time because you have an overall picture of the situation”. (administrator)
		IP 8 “There was an overall bad atmosphere. The whole team failed to function. We who were working in the ED were doing great whilst for those working with anesthesia, it was not working. We didn’t get the teamwork to function at all. There was no communication, and there were no closed loops. Nothing worked”. (registered nurse)
	Art of concise and clear information	IP 15: “Well, had I not been speaking aloud and the nurse not been speaking aloud, then I think the diagnosis would have been delayed, because we would have thought it was a stroke instead”. (physician)
	Silent communication	IP 17: “My experience is that the patient [low heart rate of 17 beats/min] is in need of this treatment, so I start connecting the patient to be ready if the decision comes. So I look up at our experienced physician and we make eye contact directly and I don’t know who said it but the decision is to perform external pacing [transcutaneous pacing]!”. (registered nurse)
Lacking management support, structure, and planning	ED considered an unsuitable place of care	IP 9: “The blood gas results improved after a couple of hours and we saw that we were doing a good job, whereas we were all in agreement that she needed to be hospitalized because she was not well [in need of advanced breathing assistance] […]. From the management via the overall responsible physician on call, down to the resident physician on call I was informed that ‘the patient will return to her home’. And I was just thunderstruck. Amazed!” (physician)
	Mismatch of available resources and excessive workload	IP 16: “It had been a terrible day for the daytime staff with a high number of patients and many severely ill patients, so they felt that they hadn’t realized that he was as wheezy as he was [non-invasive ventilator assistance]. It is possible that feeding the patients had not been prioritized. In this case, receiving food could have been beneficial [patient with diabetes and asthma] […]. He could have been assisted to inhale better, and maybe not be so oxygen-dependent. Finally, the patient ended up in the ICU, where he was intubated”. (registered nurse)
		IP 27: “Well, you don’t have time to communicate properly […]. You don’t have time to have these great briefings within the team: As a result, you know partially how we think (nursing staff) and how the physician thinks, and we can…well, talk to each other, I think”. (nurse assistant)
	Discordant views on strategies of care	IP 5: “Yes, but this is the way it is. It’s not worth being annoyed about this [nurse assistant]”. Right or wrong, but they have gotten used to the situation […]. Neurologists, in this case, don’t need to inform [the interprofessional team] about where they go, if no one cares. (registered nurse)
		IP 24: “But sometimes I think that people are a little stressed about that [statistical numbers] and maybe care a little more about the fact that everything appears to be in place, while under the blanket there is a full diaper [the situation is much worse than it appears on the surface]”. (registered nurse)
Tensions between professional role and responsibility	Gender roles and hierarchies of expertise	IP 1: “I didn’t get to have any contact with the patient because this patient turned to the male nurse all the time. Moreover, this was a rather dominant male nurse who didn’t pass the ball to me but just kept on talking. So I had a great difficulty to break into the conversation […]. (physician)
	Violation of personal and professional integrity	IP 2: “The staff could give a damn to push for the patient to get discharged. One didn’t have to put pressure on the physician from the viewpoints of the hospital bed occupational coordinator and the registered nurses.”. (physician)
Different views on interprofessional teamwork	Inadequate involvement/intrusion by the patient	IP 6:”[Oxygen] Saturation went down, down, down and I hate it when the discussion about the level of care takes place above the patients head. “Well, what is this patient actually capable of? What is their usual condition? Do they have dementia?” And then you see that the patient hears everything”. (physician)
	Personal relations and favoritism	IP 25: “He is kind of her favorite nurse. And all of us who work here know that [laughter] […]. It felt like you didn’t have any competence at all to do anything, it was just him, him, him”. (registered nurse)
	Perspective on teamwork attributes	IP 15: “There was no specific [person] who took the lead in the situation, because the cardiologist on-call, who should lead the situation, received no response to her suggestions. […] So, they didn’t listen to her or allow her to direct the care. Suggestions were discarded and directives were issued that really did not have any [receiver]. […] There was no structure in the room”. (physician)
		IP 25: “[…] But the fact that you work in the same [way] direction, I think can be important, that there is some kind of structure in how you think”. (registered nurse)
Confidence in interprofessional team members	Joint team assessments	IP 12: “It is difficult to be a nurse without a physician and it is extremely difficult to be a physician without a nurse. I think you also realize it when it becomes like this, you sort of help each other”. (registered nurse)
	Mutual need for interprofessional support	IP 7: “He said, ‘This is not ok. I will never get used to this. Never. A young beautiful human being who is executed like this. I will never get used to this’. And I thought, Wow! Well, not wow like that, of course he is human too, but this is the first time I have seen a physician stand up and be so emotionally moved that the tears just flowed.” (nurse assistant)
		IP 14: “So, it has been great…it has been good, but it has also been damn irritating that it is not always like that. One gets frustrated that it can work like this but then no one does it”. (nurse assistant)
		IP 21: “The patient wanted help. He wanted to be hospitalized. And then I walked out and told the nurses: ‘you have to go in and treat the wounds until I have spoken to the medically responsible physician’. And everyone passed on this problem to…in other words the nurses assigned it to other nurses to handle, as no one wanted to treat this patient because of the strong smell, and they didn’t really want to help him”. (physician)

IP, interview participant

### Salience of reflection

In the first category, participants referred to reflections as a learning activity, i.e., an essential factor in professional growth as individuals within interprofessional teams. The first subcategory, *self-awareness to scrutinize oneself,* describe reflections upon the ability to deliver competence to the fullest potential and giving a motivation for scrutinizing oneself (Table 4, IP19). Participants described the value of receiving feedback about personal qualities in work situations involving close collaboration. However, this was often self-initiated. Thus, the lack of feedback in close connection with the assessment of patients in the ED led to hesitation and the questioning of professional ability (Table 4, IP 18). The second subcategory, *team reflection as a way of interprofessional learning,* relates to crucial confirmation and reassurance to be able to progress and work to the best of one’s professional ability. Participants expressed disappointment with the deprioritization of standard debriefing protocols. In particular, HCPs were not able to step away from the emergency care and reflect on their conscious and unconscious actions to lift team spirit and increase motivation (Table 4, IP 7).

### Professional experience makes a difference

Knowing clinical routines and mastering professional competence were parallel ways of describing the importance of professional experience. This category encompasses three subcategories. First, *experience is a crucial component of professional practice,* refers to the level of professional experience and how adequately the patients received care. For example, introduction to the ED work environment for newly graduated HCPs or HCPs under training was described as inadequate. HCPs tied to other units with irregular work shifts in the ED lacked ED-specific skills, routines, and consistency, which hindered the work progress of the team. Thus, inexperienced members demonstrated more teamwork failures, which were considered crucial when the decisions depended on a specific profession's function (Table 4, IP 6). In addition, frequent changes of HCPs were described as contributing to the loss of teamwork spirit and diminished trust in the capacity of the team. When participants were familiar with team members’ professional skills and established routines, a sudden change of HCPs would create dissatisfaction. For the second subcategory, *expectation of professional experience,* participants described situations in which the management had fostered the expectation among newly employed HCPs that little experience was needed for the work at hand. Thus, the anticipation of other team members’ experience within a team was described as crucial and related to the specific profession and the length of clinical work experience. The subsequent teamwork was experienced as decisive depending on which team member was involved and their professional role. An imbalance in the levels of professional experience of team members represented a challenge. Nevertheless, lack of experience within a team could, if mutual awareness of communication existed, create satisfactory interprofessional teamwork. The expectation of functional teamwork emerged from work experiences with colleagues who practiced successful interprofessional teamwork (Table 4, IP 19). The *need for continuous development and training*, the third subcategory, reflects experiences regarding clinical education and simulation training. The simulation training united team members and allowed the sharing of information about ED-specific routines in acute situations and in interprofessional teamwork. Clinical training in emergency care and emergency medicine was referred to as highly important for creating a structured work flow and becoming aware of interprofessional teamwork roles (Table 4, IP 18). Lack of an introductory phase and professional training, as well as insufficient implementation of work routines (see also the category of *Management support, structure and planning*) were described as factors that delayed the assessment process for the patient. In these cases, HCPs had to navigate the work environment on their own, which generated feelings of insecurity for both the team and the individual HCP (Table 4, IP 21).

### Demanding physical and psychosocial work environment

This category highlights aspects of the possibility to perform optimal interprofessional teamwork within the ED environment. For the first subcategory, *the physical work environment,* participants described the importance of working in close physical connection to each other, enabling rapid assistance and communication. However, this was not always preferable in narrowly spaced working environments, especially when the clinical situation worsened. Colleagues with good intentions rendered assistance in such situations, even though this increased crowding, and working in a confined area became more difficult (Table 4, IP 6). Furthermore, the noisy environment with loud alarms and patient crowding in the ED disturbed the working environment (Table 4, IP 9). Second, *dealing with emotions related to stress,* participants described the challenge of balancing the onerous demands of severely ill patients with providing a satisfactory level of care for all patients. Occasionally, this led to colleagues speaking harshly and loudly (i.e., screaming) during communication with colleagues. At the same time, participants referred to the work in the ED as uniquely demanding and challenging, where they needed to conceal their feelings (Table 4, IP 2).

### Balancing communication demands

This category includes those attributes that hinder or enable interprofessional team communication, and consists of three subcategories. The first subcategory, *applying communication (tools, climate and attitudes)* relates to experiences regarding the challenge of exchanging information and, thus, balancing the demands of listening and sharing information. To balance the needs for different verbal communication tools, speaking up and closed loops and even interruptions by colleagues, were described as common and successful if used correctly (Table 4, IP 11) Participants described nursing staff and physicians who were familiar with the ED as those who established the standard ways of working. Colleagues with an unpleasant attitude were socially accepted in the ED due to their position in the internal ranking order, based on years of clinical experience and popularity. If these attributes were used in a permissive way, it was constructive. Interprofessional team members, consulting within the ED, had to be attentive to the standards so as not to create conflicts or to feel uncomfortable in their communication (Table 4, IP 8). The second subcategory, the *art of concise and clear information,* highlights the challenge of communicating precise information with the correct details on the intended occasion. Participants expressed the view that more details should be written in the medical record rather than being verbally reported to the whole team, to avoid creating confusion. In contrast, other participants pointed out the importance of ‘thinking aloud’ as a way to avoid missing any input from colleagues in the interprofessional team (Table 4, IP 15). The third subcategory, *silent communication,* relates to eye contact, facial expressions, and gestures between HCP team members. This was described by participants as something that increased or sometimes replaced verbal communication. For example, a silent question was posed through facial expressions and thoughts that could not be addressed aloud were transmitted via gestures (Table 4, IP 17). Handwritten notes about laboratory tests or prescriptions were described as silent communication that lay outside the patients’ hospital records.

### Lacking management support, structure, and planning

Participants shared experiences about the given prerequisites for the interprofessional team. This category has three subcategories. First, *ED considered an unsuitable place of care,* participants reported advanced and time-consuming healthcare situations in a suboptimal hospital location, the ED. HCPs were forced to take on unfamiliar advanced medical treatments or for which there was a lack of sufficient space, time and equipment. While the advanced medical treatment was described as being successfully implemented in the ED, with the interprofessional team, it was not however beneficial for the long-term primary patient plan. Thus, this subcategory was further described as a hazard to other waiting patients (Table 4, IP 9). Regarding the second subcategory, *mismatch of available resources and excessive workload,* participants reported experiencing direct shortages of resources, i.e., HCPs and beds, which posed a challenge to the efficiency of the interprofessional teamwork. When the number of visiting patients increased (crowding), HCPs were challenged to prioritize patients and possible interventions (Table 4, IP 16). Participants further described a lack of time to execute routines that would advance the teamwork (Table 4, IP 27). Insufficient time for care resulted in exacerbated situations and caused patients to deviate from their care plan. The third subcategory, *discordant view on strategies of care,* is concerned with experiences regarding strong confidence in traditional working routines. Situations were described in which nursing staff had become so used to the absence of physicians from the team that they no longer cared about the consequences (Table 4, IP 5). Participants further reported dysfunctional collaborations between wards and the ED, and a lack of mutual understanding of the requirements of the daily work and associated obstacles. Another example in this subcategory was the experiences of managers who directed the work from a distance, addressing statistical numbers but lacking oversight and appreciation of the quality of the bedside care and the issues that mattered on the floor of the ED (Table 4, IP 24).

### Tensions between professional role and responsibility

This category relates to experiences with crossing the line, personally and professionally. For the first subcategory, *gender roles and hierarchies of expertise*, experiences were stated regarding issues with collaboration due to gender affiliation and gender stereotypes, as well as non-dependent professional roles (Table 4, IP 1). Gender schisms were described as an accepted feature of the ED. Specifically, male colleagues were described as being more readily incorporated into the team than their female colleagues, a hierarchy in which women were ranked lower than men. The second subcategory, *violation of personal and professional integrity*, brought forth experiences related to situations in which HCPs pushed colleagues to make a rapid and unpremeditated assessment. Team members from other professions were spoken of in a disparaging way when they questioned the wisdom of certain decisions (Table 4, IP 2). Furthermore, participants described experiences where colleagues suggested a treatment but were verbally reprimanded for violating interprofessional boundaries.

### Different views on interprofessional teamwork

This category, which is concerned with experiences of how interprofessional teamwork is considered as a functional unit, comprises three subcategories. First, *inadequate involvement/intrusion by the patient,* participants described situations in which patients directly influenced the interprofessional teamwork negatively. Patients with long waiting times interrupted the teamwork with questions and a need for care. There were situations in which the patients overheard inappropriate discussions of the ambition levels of the care and delicate questions about medical restrictions (Table 4, IP 6). Second, *personal relations and favoritism*, participants described trust, based on personal relations, as being more robust in a professional teamwork. The hierarchy was less-evident and the communication was more-effective. There were situations where colleagues favored some persons over others, and this compromised the teamwork (Table 4, IP 25). In the third subcategory, *perspective on teamwork attributes*, participants referred to the team leader as someone who had the clear function of leading the team and directed all the team members in accordance with their distinct roles. To deliver adequate patient care within a reasonable timeframe, teamwork was described as being dependent upon a leader who was physically present. However, the expectations that the interviewees had of the leader were described related to a personal quality rather than the profession of the leader (Table 4, IP 15). Participants described different views of teamwork and how this affected interprofessional interactions. Moreover, participants expressed a demand of guidelines as how to collaborate interprofessionally to avoid different views on teamwork (Table 4, IP 25).

### Confidence in interprofessional team members

This category recognizes the need for mutual assistance and the impact of given or absent support. For the first subcategory, *joint team assessments,* participants described the value of assessing patients together, using the interprofessional expertise and resources within the team. Concurrently, situations in which colleagues from different professions were favored or alternatively rejected by team members were regarded as poor interprofessional teamwork. Work responsibility was individually linked and referred to as a collective interaction (Table 4, IP 12). *Mutual need for interprofessional support* constitutes the second subcategory. Participants shared experiences of vulnerability and complete dedication to the patient with their colleagues. This action encouraged confidence in team members (Table 4, IP 7). Participants described feelings of frustration when the experience of supporting colleagues could not be applied in all situations, which is when the absence of support became evident (Table 4, IP 14). In contrast, participants also shared experiences of unsupportive colleagues who acted as poor role models for their profession. These colleagues were associated with substandard treatment and negative attitudes towards patients (Table 4, IP 21).

## Discussion

Taken together, the HCPs experiences of critical incidents support the notion that interprofessional teamwork is particularly complex in the ED setting, and emphasize the idea that dysfunctional (barriers to) or functional (enablers of), communication and experience within teams are considered as important by HCPs.

Participants described the importance of having organized interprofessional team reflections, i.e., ‘debriefings’, to improve collaboration and interprofessional understanding, to gain self-awareness, and to increase professional motivation. The practice of adequate reflection in a high-risk environment was described as essential for HCP’s in the studied ED. Concerning the described experiences of tension between professional categories and protecting different areas of responsibility, reflections may work as a tool for overcoming these tensions [26]. Moreover, since the teams in this study consisted of a minimum of two professions, it was seen as beneficial to raise awareness of the other professions in the team. The reflection process was up to the HCPs to initiate, as this was not organized by management. However, this is something that the literature argues as being the responsibility of healthcare managers [27]. Second, participants considered interprofessional teamwork to be contingent upon the management establishing the appropriate prerequisites for the requested work. When management ignores its responsibility to coordinate and guide HCP teams in directions that are beneficial for patient care, the interprofessional teamwork becomes suboptimal and patient safety is placed at risk [1, 27].

Regarding the promotion of effective interprofessional communication within the interprofessional team, participants shared experiences of good communication routines in an enabling working climate. Effective communication is supported by a ‘shared mental model’ of implementing the TeamSTEPPS, described by Obenrader [28]. Especially, the perception of teamwork and communication showed to be improved in the team. Moreover, a common understanding of communication and its contribution to interprofessional teamwork is essential to advance healthcare delivery [28]. The working climate must welcome verbal and silent communications with all team members, to give space for functional development within the team [29]. This also touches on aspects of participants’ experiences with gender roles and hierarchy. HCPs face the challenge of a tension between professional roles and expertise, which have been shown to affect patient care [9, 29]. However, it has been argued that communication and understanding among professions can overcome these barriers [30]. In addition, silent communication was brought up by participants as being important. Specifically, participants emphasized the importance of ‘thinking aloud’ during acute situations, not to oversee any possible input from colleagues. The literature supports the importance of the exchange of explicit language between HCPs [12, 31]. However, interpersonal relationships, trust, and different perceptions of the same clinical situation lead to different levels of responsiveness [31], which is in accordance with the findings of the current study.

Although it has been established that interprofessional teamwork is of great importance for successful and safe care [26], many HCPs find it challenging to implement optimal teamwork. Participants expressed frustration regarding deficiencies in professional experience, competence and clinical routines. Furthermore, the existence of a schism between management expectations and clinical reality was mentioned. This reflects the findings of a previous study [32] that showed that lack of experience could be compensated by communication and support from interprofessional team members and management on all levels. The participants described hazardous situations in which patients were inappropriately treated in the ED, mainly due to management organization in relation to assessments and a lack of in-hospital beds. ED crowding due to a lack of beds or missing HCPs undermines professional authority and causes ethics-related stress, which can lead to adverse events [33].

There are certain factors that can improve and facilitate teamwork in the healthcare setting [6, 27]. The most prominent enabling factors identified in this study that influence interprofessional teamwork include a balance of several resources: support from colleagues and management; professional and clinical experience; communication; and team training. A recent study conducted by Sterner et al. [34] found an evident need amongst newly graduated nurses for support from colleagues in acute situations. The interpersonal relationship was described as a crucial factor for novice nurses to handle acute situations and improve their quality of care [34]. Those authors further described simulation-based training to bridge the experience gap in acute situations. Consequently, there is a focus on such educational support in the present study. The literature further supports the findings of the present study regarding simulation exercises and training activities that are necessary to enable decision-making, specifically for HCPs who are in leading positions (i.e., ED lead physicians) [35] and are thus responsible for interprofessional teamwork in medical care. If education and training are not offered for HCPs in specific positions, then organization and planning measures do not help if the targeted HCPs are not trained to carry out the task [35].

### Methodological considerations

The data exemplify a rich description of participants’ experiences and the situations that shaped these experiences [17, 36]. The selected participants were qualified to answer the research questions [37]. During the interviews, the participants sometimes became emotional when sharing experiences and recalling the described event. The researcher repeated information about confidentiality and offered the participants the opportunity to pause, although only one participant chose to undertake the interview over two separate occasions. The preunderstanding of the phenomenon and context was discussed in the research group to maintain objectivity [38].

There is a challenge associated with strictly dichotomizing enablers and barriers to interprofessional teamwork, which we dealt with by allowing one category to have both denominations, through different aspects. The complexity in the ED environment is evident and contributes to the double-sided described experiences (factors that are both enablers and barriers) in relation to interprofessional teamwork.

### Limitations

The study has some limitations. First, we cannot exclude the possibility of bias from the purposeful selection of participants, although we made an effort to avoid such bias by applying careful sampling of maximum variation. Second, all the participants were asked to recall their retrospective experiences and give detailed descriptions of critical incidents in relation to interprofessional teamwork. While memories may change when recalling events, the presented experiences were readily provided in detail and connected to a specific time and place [36]. Third, critical incidents should be combined with other data, such as observations, to support the described experiences [16]. Our study did not use such supporting data and considered to have generated a generous amount of critical incidents and rich textual data [17]. The rich material made the researchers aware of the potential risk of omitting details, which motivated an in-depth analysis of the material. Finally, this project evaluated only one ED, which might limit transferability of the findings. In this context, it would be interesting to investigate the effects of team size, composition, and rural or urban setting.

## Conclusion

The findings of this study suggest that ED-specific communication and professional and clinical experience are essential factors in interprofessional teamwork in relation to critical incidents in the ED. Apart from communication and experience, management plays a central role in enabling interprofessional teamwork through organizing the ED work and setting appropriate expectations with the HCPs. The influences that management has on the physical and psychosocial work environment are important for optimal interprofessional teamwork and for the general working climate. In summary, key factors are identified to enable interprofessional teamwork to manage critical incidents in the complex working environment of the ED. This study is of relevance to clinicians and managers in raising awareness of communication behaviors in relation to critical incidents in the ED.

## Supplementary Information

**Additional file 1**. Supplementary file COREQ (COnsolidated criteria for REporting Qualitative research) Checklist.

## Data Availability

The datasets generated and/or analyzed during the current study are not publicly available due to the protection of confidentiality for the interview participants. However, the data are available from the corresponding author upon reasonable request.

## Abbreviations

HCP: Healthcare professional
ED: Emergency department
CIT: Critical incident technique
